# (*E*)-Methyl *N*′-(3-hy­droxy­benzyl­idene)hydrazinecarboxyl­ate dihydrate

**DOI:** 10.1107/S1600536811035689

**Published:** 2011-09-14

**Authors:** Wei-Wei Li, Tie-Ming Yu, Lu-Ping Lv, Xian-Chao Hu

**Affiliations:** a Linjiang College, Hangzhou Vocational and Technical College, Hangzhou 310018, People’s Republic of China; b College of Chemical Engineering and Materials Science, Zhejiang University of Technology, Hangzhou 310014, People’s Republic of China; c Research Center of Analysis and Measurement, Zhejiang University of Technology, Hangzhou 310014, People’s Republic of China

## Abstract

The title compound, C_9_H_10_N_2_O_3_·2H_2_O, crystallizes with two organic mol­ecules and four water mol­ecules in the asymmetric unit. Both organic mol­ecules adopt a *trans* conformation with respect to the C=N bond and are close to planar [dihedral angles between the side chain and the aromatic ring = 9.34 (8) and 4.96 (8)°]. In the crystal, the components are linked into three-dimensional network by N—H⋯O and O—H⋯O hydrogen bonds.

## Related literature

For background to benzaldehyde­hydrazone derivatives, see: Parashar *et al.* (1988 ▶); Hadjoudis *et al.* (1987 ▶); Borg *et al.* (1999 ▶). For a related structure, see: Shang *et al.* (2007 ▶).
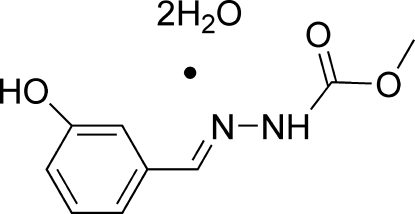


## Experimental

### 

#### Crystal data

C_9_H_10_N_2_O_3_·2H_2_O
*M* *_r_* = 230.22
Monoclinic, 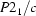

*a* = 11.7316 (16) Å
*b* = 20.785 (3) Å
*c* = 9.5259 (16) Å
β = 99.675 (3)°
*V* = 2289.7 (6) Å^3^
*Z* = 8
Mo *K*α radiation
μ = 0.11 mm^−1^
*T* = 223 K
0.18 × 0.17 × 0.15 mm

#### Data collection

Bruker SMART CCD diffractometer
Absorption correction: multi-scan (*SADABS*; Bruker, 2002 ▶) *T*_min_ = 0.977, *T*_max_ = 0.989
19583 measured reflections
4438 independent reflections
3346 reflections with *I* > 2σ(*I*)
*R*_int_ = 0.023

#### Refinement

*R*[*F* ^2^ > 2σ(*F* ^2^)] = 0.040
*wR*(*F* ^2^) = 0.117
*S* = 1.03
4438 reflections
326 parameters
H atoms treated by a mixture of independent and constrained refinement
Δρ_max_ = 0.22 e Å^−3^
Δρ_min_ = −0.14 e Å^−3^

### 

Data collection: *SMART* (Bruker, 2002 ▶); cell refinement: *SAINT* (Bruker, 2002 ▶); data reduction: *SAINT*; program(s) used to solve structure: *SHELXS97* (Sheldrick, 2008 ▶); program(s) used to refine structure: *SHELXL97* (Sheldrick, 2008 ▶); molecular graphics: *SHELXTL* (Sheldrick, 2008 ▶); software used to prepare material for publication: *SHELXTL*.

## Supplementary Material

Crystal structure: contains datablock(s) I, global. DOI: 10.1107/S1600536811035689/hb6397sup1.cif

Structure factors: contains datablock(s) I. DOI: 10.1107/S1600536811035689/hb6397Isup2.hkl

Additional supplementary materials:  crystallographic information; 3D view; checkCIF report

## Figures and Tables

**Table 1:** Hydrogen-bond geometry (Å, °)

*D*—H⋯*A*	*D*—H	H⋯*A*	*D*⋯*A*	*D*—H⋯*A*
O1*W*—H1*B*⋯O2*W*	0.85 (3)	2.02 (3)	2.859 (2)	169 (2)
O1*W*—H1*A*⋯O2*W*^i^	0.86 (3)	1.95 (3)	2.809 (3)	172 (3)
N2—H2⋯O6^ii^	0.86	2.07	2.9278 (17)	171
O2*W*—H2*A*⋯O4^iii^	1.02 (3)	1.88 (3)	2.893 (2)	171 (3)
O2*W*—H2*B*⋯O1	0.81 (3)	2.23 (3)	2.9102 (18)	141 (3)
O2*W*—H2*B*⋯N1	0.81 (3)	2.59 (3)	3.322 (2)	149 (3)
O3*W*—H3*A*⋯O4^iv^	0.88 (3)	2.15 (3)	2.877 (2)	139 (3)
O3*W*—H3*B*⋯O1^v^	0.95 (3)	1.90 (3)	2.832 (2)	168 (3)
O3—H3*W*⋯O1*W*	0.82	1.93	2.6617 (19)	147
O4*W*—H4*B*⋯O3*W*^iii^	0.87 (3)	2.00 (3)	2.864 (2)	172 (2)
O4*W*—H4*A*⋯O3*W*^vi^	0.82 (4)	2.20 (4)	3.013 (3)	170 (3)
N4—H4*N*⋯O3^vii^	0.86	2.10	2.9509 (18)	169
O6—H6⋯O4*W*	0.92 (3)	1.75 (3)	2.665 (2)	176 (2)

Symmetry codes: (i) 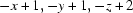; (ii) 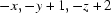; (iii) 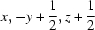; (iv) 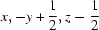; (v) 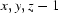; (vi) 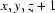; (vii) 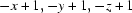.

**Fig. 1.**
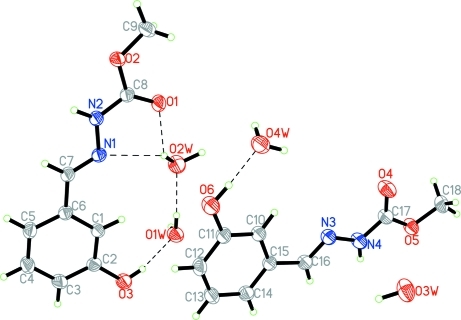
Molecular structure of (I), showing 30% probability displacement ellipsoids. Dashed lines represent hydrogen bonds.

**Fig. 2.**
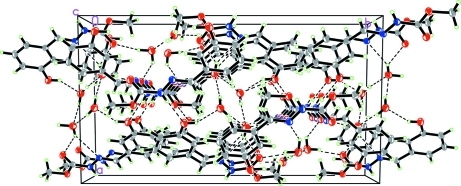
Crystal packing of the title compound, viewed approximately down the c axis. Dashed lines indicate hydrogen bonds. H atoms not intervening in H-bonding were eliminated for clarity.

## supplementary crystallographic information

### Comment

Benzaldehydehydrazone derivatives have received considerable attentions for a long time due to their pharmacological activity (Parashar *et al.*, 1988) and their photochromic properties(Hadjoudis *et al.*, 1987). Meanwhile, it's an important intermidiate of 1,3,4-oxadiazoles, which have been reported to be versatile compounds with many properties(Borg *et al.*, 1999). As a further investigation of this type of derivatives, we report herein the crystal structure of the title compound, (I).

The title compound, C_9_H_10_N_2_O_3_ .2H_2_O, crystallizes with two very similar independent molecules in the asymmetric unit. Each independent molecule adopts a *trans* configuration with respect to the C═N bond. The N1/N2/O1/O2/C7-C9 and N3/N4/O4/O5/C16-C18 planes form dihedral angles of 9.34 (8)° and 4.96 (8)°, respectively, with the C1—C6 and C10—C15 planes. The bond lengths and angles of the main molecule agree with those observed for (E)-Methyl N'-(4-hydroxybenzylidene)hydrazinecarboxylate (Shang *et al.*, 2007).

In the crystal structure, Intramolecular O—H···N and O—H···O hydrogen bonds are observed in each independent molecule. molecules are linked into three-dimensional network by N—H···O and O—H···Ohydrogen bonds (Table 1, Fig.2).

### Experimental

3-Hydroxybenzaldehyde (1.22g, 0.01mol) and methyl hydrazinecarboxylate(0.9g, 0.01mol) were dissolved in stirred methanol (30ml) and left for 2h at room temperature. The resulting solid was filtered off and recrystallized from ethanol to give the title compound in 90% yield.  Colourless blocks of (I) were obtained by slow evaporation of a ethanol solution at room temperature (m.p. 418-421 K).

### Refinement

H atoms of the water molecule were located in a difference map and were refined with O-H distances restrained to 0.81 (3) Å, 0.82 (3) Å, 0.85 (3) Å, 0.86 (3) Å, 0.87 (3) Å,0.88 (3) Å, 0.95 (3) Åand 1.02 (3) Å, H atoms were included in the riding model approximation with N-H = 0.86Å and O-H=0.82Å. C-bound H atoms were positioned geometrically (C-H = 0.93Å and 0.96Å) and refined using a riding model,  with U_iso_(H) = 1.2-1.5U_eq_(C).

### Crystal data

**Table:**

C_9_H_10_N_2_O_3_·2H_2_O	*F*(000) = 976
*M**_r_* = 230.22	*D*_x_ = 1.336 Mg m^−^^3^
Monoclinic, *P*2_1_/*c*	Mo *K*α radiation, λ = 0.71073 Å
Hall symbol: -P 2ybc	Cell parameters from 4438 reflections
*a* = 11.7316 (16) Å	θ = 1.6–26.0°
*b* = 20.785 (3) Å	µ = 0.11 mm^−^^1^
*c* = 9.5259 (16) Å	*T* = 223 K
β = 99.675 (3)°	Block, colourless
*V* = 2289.7 (6) Å^3^	0.18 × 0.17 × 0.15 mm
*Z* = 8	

### Data collection

**Table:**

Bruker SMART CCD diffractometer	4438 independent reflections
Radiation source: fine-focus sealed tube	3346 reflections with *I* > 2σ(*I*)
graphite	*R*_int_ = 0.023
φ and ω scans	θ_max_ = 26.0°, θ_min_ = 1.8°
Absorption correction: multi-scan (*SADABS*; Bruker, 2002)	*h* = −13→14
*T*_min_ = 0.977, *T*_max_ = 0.989	*k* = −24→25
19583 measured reflections	*l* = −11→11

### Refinement

**Table:**

Refinement on *F*^2^	Secondary atom site location: difference Fourier map
Least-squares matrix: full	Hydrogen site location: inferred from neighbouring sites
*R*[*F*^2^ > 2σ(*F*^2^)] = 0.040	H atoms treated by a mixture of independent and constrained refinement
*wR*(*F*^2^) = 0.117	*w* = 1/[σ^2^(*F*_o_^2^) + (0.0525*P*)^2^ + 0.6042*P*] where *P* = (*F*_o_^2^ + 2*F*_c_^2^)/3
*S* = 1.03	(Δ/σ)_max_ < 0.001
4438 reflections	Δρ_max_ = 0.22 e Å^−^^3^
326 parameters	Δρ_min_ = −0.14 e Å^−^^3^
0 restraints	Extinction correction: *SHELXL*, Fc^*^=kFc[1+0.001xFc^2^λ^3^/sin(2θ)]^-1/4^
Primary atom site location: structure-invariant direct methods	Extinction coefficient: 0.0121 (13)

### Special details

**Table:**

Geometry. All esds (except the esd in the dihedral angle between two l.s. planes) are estimated using the full covariance matrix. The cell esds are taken into account individually in the estimation of esds in distances, angles and torsion angles; correlations between esds in cell parameters are only used when they are defined by crystal symmetry. An approximate (isotropic) treatment of cell esds is used for estimating esds involving l.s. planes.
Refinement. Refinement of F^2^ against ALL reflections. The weighted R-factor wR and goodness of fit S are based on F^2^, conventional R-factors R are based on F, with F set to zero for negative F^2^. The threshold expression of F^2^ > 2sigma(F^2^) is used only for calculating R-factors(gt) etc. and is not relevant to the choice of reflections for refinement. R-factors based on F^2^ are statistically about twice as large as those based on F, and R- factors based on ALL data will be even larger.

### Fractional atomic coordinates and isotropic or equivalent isotropic displacement parameters (Å^2^)

**Table:**

	*x*	*y*	*z*	*U*_iso_*/*U*_eq_	
C1	0.24171 (13)	0.62113 (8)	0.93235 (17)	0.0448 (4)	
H1	0.2511	0.5777	0.9139	0.054*	
C2	0.29711 (14)	0.66693 (8)	0.86354 (17)	0.0482 (4)	
C3	0.28141 (16)	0.73158 (9)	0.8879 (2)	0.0573 (5)	
H3	0.3184	0.7624	0.8410	0.069*	
C4	0.21077 (18)	0.74998 (9)	0.9821 (2)	0.0646 (5)	
H4	0.1997	0.7935	0.9981	0.078*	
C5	0.15620 (16)	0.70477 (8)	1.0530 (2)	0.0572 (5)	
H5	0.1088	0.7178	1.1168	0.069*	
C6	0.17198 (13)	0.64001 (8)	1.02913 (17)	0.0445 (4)	
C7	0.11467 (14)	0.59292 (8)	1.10740 (17)	0.0473 (4)	
H7	0.0626	0.6075	1.1640	0.057*	
C8	0.08718 (13)	0.43208 (8)	1.18676 (17)	0.0445 (4)	
C9	0.02974 (17)	0.33892 (8)	1.2976 (2)	0.0630 (5)	
H9A	−0.0218	0.3263	1.3609	0.095*	
H9B	0.1077	0.3282	1.3394	0.095*	
H9C	0.0093	0.3167	1.2086	0.095*	
C10	0.24610 (13)	0.40621 (8)	0.55031 (17)	0.0480 (4)	
H10	0.2391	0.3631	0.5735	0.058*	
C11	0.17966 (14)	0.45186 (8)	0.60274 (19)	0.0522 (4)	
C12	0.19031 (17)	0.51613 (9)	0.5701 (2)	0.0607 (5)	
H12	0.1448	0.5469	0.6051	0.073*	
C13	0.26877 (18)	0.53409 (9)	0.4855 (2)	0.0644 (5)	
H13	0.2769	0.5774	0.4643	0.077*	
C14	0.33570 (16)	0.48887 (9)	0.43165 (19)	0.0570 (5)	
H14	0.3887	0.5016	0.3746	0.068*	
C15	0.32374 (14)	0.42458 (8)	0.46271 (17)	0.0467 (4)	
C16	0.39374 (15)	0.37710 (8)	0.40232 (19)	0.0539 (4)	
H16	0.4485	0.3914	0.3493	0.065*	
C17	0.44785 (15)	0.21513 (8)	0.36295 (19)	0.0533 (4)	
C18	0.52822 (17)	0.12027 (9)	0.2861 (2)	0.0629 (5)	
H18A	0.5858	0.1071	0.2314	0.094*	
H18B	0.4541	0.1039	0.2426	0.094*	
H18C	0.5475	0.1037	0.3812	0.094*	
N1	0.13314 (11)	0.53299 (6)	1.10118 (14)	0.0444 (3)	
N2	0.07182 (11)	0.49575 (6)	1.18140 (14)	0.0483 (3)	
H2	0.0235	0.5134	1.2282	0.058*	
N3	0.38247 (12)	0.31715 (7)	0.41971 (16)	0.0534 (4)	
N4	0.45443 (13)	0.27907 (7)	0.35476 (18)	0.0629 (4)	
H4N	0.5036	0.2965	0.3089	0.075*	
O1	0.15029 (10)	0.40159 (6)	1.12288 (14)	0.0586 (3)	
O2	0.02087 (10)	0.40691 (5)	1.27386 (13)	0.0539 (3)	
O1W	0.45469 (16)	0.53255 (7)	0.82064 (18)	0.0679 (4)	
O3	0.36825 (11)	0.65024 (6)	0.76926 (14)	0.0668 (4)	
H3W	0.3707	0.6109	0.7630	0.100*	
O2W	0.33906 (13)	0.44786 (8)	0.98696 (18)	0.0720 (4)	
O4	0.38368 (12)	0.18520 (6)	0.42559 (17)	0.0732 (4)	
O3W	0.19045 (16)	0.27222 (8)	0.05155 (19)	0.0845 (5)	
O5	0.52396 (11)	0.18889 (6)	0.29060 (15)	0.0629 (4)	
O4W	0.10070 (16)	0.31023 (8)	0.7479 (2)	0.0787 (5)	
O6	0.10057 (13)	0.43537 (7)	0.68672 (17)	0.0761 (4)	
H4A	0.132 (3)	0.2983 (17)	0.827 (4)	0.141 (15)*	
H1A	0.518 (2)	0.5347 (13)	0.881 (3)	0.102 (10)*	
H1B	0.413 (2)	0.5075 (13)	0.861 (3)	0.094 (8)*	
H4B	0.135 (2)	0.2862 (13)	0.692 (3)	0.092 (8)*	
H2B	0.279 (2)	0.4539 (13)	1.017 (3)	0.106 (9)*	
H2A	0.347 (3)	0.4003 (17)	0.965 (3)	0.139 (11)*	
H3A	0.263 (3)	0.2683 (15)	0.039 (3)	0.126 (11)*	
H3B	0.187 (2)	0.3149 (15)	0.085 (3)	0.120 (10)*	
H6	0.103 (2)	0.3920 (13)	0.705 (2)	0.094 (8)*	

### Atomic displacement parameters (Å^2^)

**Table:**

	*U*^11^	*U*^22^	*U*^33^	*U*^12^	*U*^13^	*U*^23^
C1	0.0445 (8)	0.0408 (9)	0.0520 (9)	0.0018 (7)	0.0162 (7)	0.0025 (7)
C2	0.0450 (9)	0.0510 (10)	0.0519 (9)	0.0020 (7)	0.0176 (7)	0.0062 (7)
C3	0.0626 (11)	0.0454 (10)	0.0690 (11)	−0.0062 (8)	0.0262 (9)	0.0070 (8)
C4	0.0798 (13)	0.0409 (10)	0.0801 (13)	−0.0042 (9)	0.0336 (11)	−0.0047 (9)
C5	0.0653 (11)	0.0479 (10)	0.0654 (11)	−0.0014 (8)	0.0313 (9)	−0.0075 (8)
C6	0.0411 (8)	0.0458 (9)	0.0485 (9)	−0.0020 (7)	0.0128 (7)	−0.0003 (7)
C7	0.0462 (9)	0.0467 (10)	0.0540 (9)	−0.0012 (7)	0.0225 (7)	−0.0019 (7)
C8	0.0418 (8)	0.0441 (9)	0.0501 (9)	−0.0038 (7)	0.0152 (7)	−0.0024 (7)
C9	0.0709 (12)	0.0434 (10)	0.0791 (13)	−0.0057 (8)	0.0253 (10)	0.0067 (9)
C10	0.0461 (9)	0.0441 (9)	0.0579 (10)	0.0014 (7)	0.0200 (8)	0.0006 (7)
C11	0.0489 (9)	0.0520 (10)	0.0607 (10)	0.0027 (7)	0.0236 (8)	−0.0013 (8)
C12	0.0687 (12)	0.0491 (11)	0.0712 (12)	0.0085 (9)	0.0317 (10)	−0.0057 (8)
C13	0.0806 (13)	0.0430 (10)	0.0763 (12)	0.0006 (9)	0.0326 (11)	0.0001 (9)
C14	0.0634 (11)	0.0507 (10)	0.0633 (11)	−0.0012 (8)	0.0292 (9)	0.0035 (8)
C15	0.0460 (9)	0.0470 (9)	0.0500 (9)	0.0023 (7)	0.0168 (7)	0.0001 (7)
C16	0.0557 (10)	0.0497 (11)	0.0637 (11)	0.0022 (8)	0.0316 (9)	0.0034 (8)
C17	0.0485 (9)	0.0490 (10)	0.0680 (11)	0.0027 (8)	0.0259 (8)	0.0024 (8)
C18	0.0634 (11)	0.0483 (11)	0.0799 (13)	0.0027 (8)	0.0203 (10)	−0.0109 (9)
N1	0.0421 (7)	0.0455 (8)	0.0493 (7)	−0.0037 (6)	0.0184 (6)	0.0001 (6)
N2	0.0490 (7)	0.0413 (8)	0.0618 (8)	−0.0004 (6)	0.0296 (7)	−0.0002 (6)
N3	0.0511 (8)	0.0493 (9)	0.0671 (9)	0.0047 (6)	0.0307 (7)	0.0012 (7)
N4	0.0639 (9)	0.0461 (9)	0.0916 (11)	0.0041 (7)	0.0501 (9)	0.0029 (8)
O1	0.0615 (7)	0.0477 (7)	0.0744 (8)	0.0030 (6)	0.0338 (6)	−0.0050 (6)
O2	0.0568 (7)	0.0418 (6)	0.0699 (8)	−0.0023 (5)	0.0302 (6)	0.0034 (5)
O1W	0.0705 (10)	0.0576 (9)	0.0822 (10)	0.0064 (7)	0.0321 (9)	0.0102 (7)
O3	0.0772 (9)	0.0521 (7)	0.0843 (9)	0.0051 (6)	0.0516 (7)	0.0114 (6)
O2W	0.0593 (8)	0.0651 (10)	0.1011 (11)	−0.0063 (7)	0.0406 (8)	−0.0012 (8)
O4	0.0724 (9)	0.0517 (8)	0.1089 (11)	−0.0003 (6)	0.0542 (8)	0.0060 (7)
O3W	0.0890 (11)	0.0631 (10)	0.1173 (13)	−0.0099 (8)	0.0631 (10)	−0.0202 (9)
O5	0.0653 (8)	0.0459 (7)	0.0876 (9)	0.0016 (6)	0.0423 (7)	−0.0042 (6)
O4W	0.0942 (12)	0.0573 (9)	0.0935 (12)	0.0052 (8)	0.0418 (11)	0.0071 (9)
O6	0.0803 (10)	0.0565 (9)	0.1084 (12)	0.0107 (7)	0.0646 (9)	0.0068 (8)

### Geometric parameters (Å, °)

**Table:**

C1—C2	1.379 (2)	C13—C14	1.378 (3)
C1—C6	1.388 (2)	C13—H13	0.9300
C1—H1	0.9300	C14—C15	1.381 (2)
C2—O3	1.3695 (19)	C14—H14	0.9300
C2—C3	1.381 (2)	C15—C16	1.462 (2)
C3—C4	1.374 (2)	C16—N3	1.267 (2)
C3—H3	0.9300	C16—H16	0.9300
C4—C5	1.376 (2)	C17—O4	1.209 (2)
C4—H4	0.9300	C17—O5	1.3329 (19)
C5—C6	1.383 (2)	C17—N4	1.334 (2)
C5—H5	0.9300	C18—O5	1.428 (2)
C6—C7	1.461 (2)	C18—H18A	0.9600
C7—N1	1.267 (2)	C18—H18B	0.9600
C7—H7	0.9300	C18—H18C	0.9600
C8—O1	1.2132 (18)	N1—N2	1.3731 (17)
C8—N2	1.335 (2)	N2—H2	0.8600
C8—O2	1.3357 (18)	N3—N4	1.3776 (18)
C9—O2	1.432 (2)	N4—H4N	0.8600
C9—H9A	0.9600	O1W—H1A	0.86 (3)
C9—H9B	0.9600	O1W—H1B	0.85 (3)
C9—H9C	0.9600	O3—H3W	0.8200
C10—C11	1.374 (2)	O2W—H2B	0.81 (3)
C10—C15	1.388 (2)	O2W—H2A	1.02 (3)
C10—H10	0.9300	O3W—H3A	0.88 (3)
C11—O6	1.367 (2)	O3W—H3B	0.95 (3)
C11—C12	1.382 (3)	O4W—H4A	0.82 (4)
C12—C13	1.373 (3)	O4W—H4B	0.87 (3)
C12—H12	0.9300	O6—H6	0.92 (3)
			
C2—C1—C6	119.88 (15)	C11—C12—H12	120.3
C2—C1—H1	120.1	C12—C13—C14	120.89 (17)
C6—C1—H1	120.1	C12—C13—H13	119.6
O3—C2—C1	121.65 (15)	C14—C13—H13	119.6
O3—C2—C3	118.03 (14)	C13—C14—C15	119.70 (16)
C1—C2—C3	120.31 (15)	C13—C14—H14	120.2
C4—C3—C2	119.52 (16)	C15—C14—H14	120.2
C4—C3—H3	120.2	C14—C15—C10	119.71 (15)
C2—C3—H3	120.2	C14—C15—C16	119.00 (15)
C3—C4—C5	120.79 (17)	C10—C15—C16	121.30 (15)
C3—C4—H4	119.6	N3—C16—C15	122.31 (15)
C5—C4—H4	119.6	N3—C16—H16	118.8
C4—C5—C6	119.84 (16)	C15—C16—H16	118.8
C4—C5—H5	120.1	O4—C17—O5	124.86 (17)
C6—C5—H5	120.1	O4—C17—N4	126.06 (16)
C5—C6—C1	119.63 (15)	O5—C17—N4	109.08 (14)
C5—C6—C7	118.84 (14)	O5—C18—H18A	109.5
C1—C6—C7	121.52 (15)	O5—C18—H18B	109.5
N1—C7—C6	122.52 (14)	H18A—C18—H18B	109.5
N1—C7—H7	118.7	O5—C18—H18C	109.5
C6—C7—H7	118.7	H18A—C18—H18C	109.5
O1—C8—N2	126.05 (14)	H18B—C18—H18C	109.5
O1—C8—O2	125.11 (15)	C7—N1—N2	114.72 (13)
N2—C8—O2	108.84 (13)	C8—N2—N1	119.99 (12)
O2—C9—H9A	109.5	C8—N2—H2	120.0
O2—C9—H9B	109.5	N1—N2—H2	120.0
H9A—C9—H9B	109.5	C16—N3—N4	114.91 (14)
O2—C9—H9C	109.5	C17—N4—N3	120.01 (14)
H9A—C9—H9C	109.5	C17—N4—H4N	120.0
H9B—C9—H9C	109.5	N3—N4—H4N	120.0
C11—C10—C15	119.87 (16)	C8—O2—C9	116.76 (13)
C11—C10—H10	120.1	H1A—O1W—H1B	103 (2)
C15—C10—H10	120.1	C2—O3—H3W	109.5
O6—C11—C10	121.45 (16)	H2B—O2W—H2A	109 (3)
O6—C11—C12	118.05 (15)	H3A—O3W—H3B	103 (3)
C10—C11—C12	120.50 (15)	C17—O5—C18	117.01 (14)
C13—C12—C11	119.32 (16)	H4A—O4W—H4B	102 (3)
C13—C12—H12	120.3	C11—O6—H6	110.8 (15)
			
C6—C1—C2—O3	178.82 (15)	C13—C14—C15—C10	−1.3 (3)
C6—C1—C2—C3	−1.5 (3)	C13—C14—C15—C16	178.77 (18)
O3—C2—C3—C4	−179.89 (17)	C11—C10—C15—C14	1.6 (3)
C1—C2—C3—C4	0.4 (3)	C11—C10—C15—C16	−178.51 (17)
C2—C3—C4—C5	0.5 (3)	C14—C15—C16—N3	−175.88 (18)
C3—C4—C5—C6	−0.3 (3)	C10—C15—C16—N3	4.2 (3)
C4—C5—C6—C1	−0.8 (3)	C6—C7—N1—N2	−179.94 (14)
C4—C5—C6—C7	179.01 (17)	O1—C8—N2—N1	−2.0 (3)
C2—C1—C6—C5	1.7 (2)	O2—C8—N2—N1	178.36 (13)
C2—C1—C6—C7	−178.10 (15)	C7—N1—N2—C8	−177.79 (15)
C5—C6—C7—N1	−173.42 (17)	C15—C16—N3—N4	179.48 (16)
C1—C6—C7—N1	6.4 (3)	O4—C17—N4—N3	−1.1 (3)
C15—C10—C11—O6	178.56 (17)	O5—C17—N4—N3	178.98 (15)
C15—C10—C11—C12	−0.7 (3)	C16—N3—N4—C17	−178.25 (18)
O6—C11—C12—C13	−179.77 (19)	O1—C8—O2—C9	2.9 (2)
C10—C11—C12—C13	−0.5 (3)	N2—C8—O2—C9	−177.42 (15)
C11—C12—C13—C14	0.8 (3)	O4—C17—O5—C18	0.8 (3)
C12—C13—C14—C15	0.1 (3)	N4—C17—O5—C18	−179.25 (16)

### Hydrogen-bond geometry (Å, °)

**Table:**

*D*—H···*A*	*D*—H	H···*A*	*D*···*A*	*D*—H···*A*
O1W—H1B···O2W	0.85 (3)	2.02 (3)	2.859 (2)	169 (2)
O1W—H1A···O2W^i^	0.86 (3)	1.95 (3)	2.809 (3)	172 (3)
N2—H2···O6^ii^	0.86	2.07	2.9278 (17)	171.
O2W—H2A···O4^iii^	1.02 (3)	1.88 (3)	2.893 (2)	171 (3)
O2W—H2B···O1	0.81 (3)	2.23 (3)	2.9102 (18)	141 (3)
O2W—H2B···N1	0.81 (3)	2.59 (3)	3.322 (2)	149 (3)
O3W—H3A···O4^iv^	0.88 (3)	2.15 (3)	2.877 (2)	139 (3)
O3W—H3B···O1^v^	0.95 (3)	1.90 (3)	2.832 (2)	168 (3)
O3—H3W···O1W	0.82	1.93	2.6617 (19)	147.
O4W—H4B···O3W^iii^	0.87 (3)	2.00 (3)	2.864 (2)	172 (2)
O4W—H4A···O3W^vi^	0.82 (4)	2.20 (4)	3.013 (3)	170 (3)
N4—H4N···O3^vii^	0.86	2.10	2.9509 (18)	169.
O6—H6···O4W	0.92 (3)	1.75 (3)	2.665 (2)	176 (2)

Symmetry codes: (i) −*x*+1, −*y*+1, −*z*+2; (ii) −*x*, −*y*+1, −*z*+2; (iii) *x*, −*y*+1/2, *z*+1/2; (iv) *x*, −*y*+1/2, *z*−1/2; (v) *x*, *y*, *z*−1; (vi) *x*, *y*, *z*+1; (vii) −*x*+1, −*y*+1, −*z*+1.
